# The Properties of Microwave-Assisted Synthesis of Metal–Organic Frameworks and Their Applications

**DOI:** 10.3390/nano13020352

**Published:** 2023-01-15

**Authors:** Pham Thi Phan, Jeongsoo Hong, Ngo Tran, Thi Hoa Le

**Affiliations:** 1 Faculty of Food Science and Engineering, Lac Hong University, Bien Hoa 810000, Vietnam; 2 Department of Electrical Engineering, Gachon University, 1342 Seongnamdaero, Seongnam 13120, Republic of Korea; 3 Institute of Research and Development, Duy Tan University, Da Nang 550000, Vietnam; 4 Faculty of Natural Sciences, Duy Tan University, Da Nang 550000, Vietnam; 5 Department of Chemical and Biological Engineering, Gachon University, Seongnam 13120, Republic of Korea

**Keywords:** metal–organic framework (MOF), microwave (MW)-assisted synthesis, kinetics, porosity, crystal, aggregation

## Abstract

Metal–organic frameworks (MOF) are a class of porous materials with various functions based on their host-guest chemistry. Their selectivity, diffusion kinetics, and catalytic activity are influenced by their design and synthetic procedure. The synthesis of different MOFs has been of considerable interest during the past decade thanks to their various applications in the arena of sensors, catalysts, adsorption, and electronic devices. Among the different techniques for the synthesis of MOFs, such as the solvothermal, sonochemical, ionothermal, and mechanochemical processes, microwave-assisted synthesis has clinched a significant place in MOF synthesis. The main assets of microwave-assisted synthesis are the short reaction time, the fast rate of nucleation, and the modified properties of MOFs. The review encompasses the development of the microwave-assisted synthesis of MOFs, their properties, and their applications in various fields.

## 1. Introduction

The energy storage technologies for future generations depend on various rudiments. Among these, the metal–organic framework (MOF) is considered one of the most significant entrants. MOFs are porous and comprise organic linkers and metal nodes. The structural and chemical properties of their particles, such as conductivity, stability, porosity, and morphology, can be manipulated by applying different synthetic procedures [1,2,3,4,5]. Thus, electrochemical limitations can be overcome and the material properties of diverse devices used for energy storage can be optimised on the basis of their synthetic adaptability. They have been extensively used in designing functional materials with specific morphologies and expected chemical compositions because they can be easily tuned. Different MOF derivatives have been used for designing energy storage devices, biomedical material, and electrocatalysts: porous compounds, nanocomposites, and metallic compounds [6,7,8,9,10].

The interdisciplinary research on MOFs evolved around 2 decades ago. In the early 1990s, coordination chemists working on crystal engineering designed the porous structure by assembling inorganic and organic building blocks [11]. Scientists working on zeolites incorporated organic molecules as reactants in the framework at elevated temperatures [12]. Different synthetic methods with different reaction temperatures have been employed for the synthesis of MOFs. Mechanochemical and electrochemical syntheses are used for the introduction of linkers within the MOF by coordination chemists, while microwave synthesis, solvothermal, and steam-assisted reactions are studied by chemists working on zeolites [13].

Moreover, the research was also directed towards the introduction of the microwave (MW)-assisted synthesis of MOFs for enhancing the stability of the MOF structure. The morphology of MOFs depends on the synthesis method in that the same reaction mixture leads to different MOFs depending on various parameters: reaction time, morphology, yield, and particle size. Because the different properties of MOFs can be characterised by multiple techniques, the aforementioned parameters hindering the performance of an electronic device can be easily identified. The properties of MOFs are significantly affected by covalent bonding [14].

Thus, the design of a highly porous structure has been emphasised for its application in adsorption, gas storage, catalysts, and electrical components. This development of porous structures opens the realm for the future development of MOFs with augmented performance.

The conventional synthetic methods for the preparation of MOFs were developed in three major stages:

1. The initial method was carried out mainly through diffusion or slow evaporation for a long duration (days to months) to produce large single crystals.
2. This was followed by the application of a solvothermal process, which is a conventional method for the synthesis of zeolites. Although the solvothermal process decreased the synthesis time from months to some days, the formation of microcrystalline powders became more prominent. Viscous gels or amorphous solids are generally obtained through solvothermal synthesis, particularly for Hf-MOFs. Hence, the method required huge manipulations for the formation of a single crystal.
3. Another commonly used technique for the preparation of MOFs is a hydrothermal synthesis, which also requires a long duration for the reaction to complete, extending from multiple hours to days. Thus, the crystal engineers had to find an efficient method for decreasing the synthesis time of MOFs along with the formation of structured crystals.

Microwave (MW)-assisted synthesis has become an alternative method for both organic and inorganic materials as it is associated with a reduced amount of time required for the synthesis of porous materials such as MOFs from days to minutes [15]. The crystallisation procedure for the formation of porous materials is shortened under MW thanks to the faster rate of nucleation. Conventional MOF synthesis requires different solvents, which are highly toxic and can be avoided in the MW-assisted synthesis. Moreover, the properties and function of MOFs obtained by MW synthesis are highly improved, thereby proving the significance of the procedure for future endeavours [16]. A solution can be uniformly and rapidly heated under MW fields by coupling through conduction and polarisation with molecules within the solution. The process by which ions and free charge carriers move along an electric field is termed “conduction”, and the process by which the dipoles align along an oscillating field is termed “polarisation”. The rotation of dipoles during polarisation causes collision among molecules and atoms in the solution, resulting in dielectric heating. Similarly, the collisions of atoms and molecules due to conduction generate ohmic heating. MW heating is shown mathematically in Equation (1):

(1) $$\varepsilon =\varepsilon^{\prime }-j\left(\varepsilon^{\prime \prime }+\frac{\sigma }{\omega }\right)ӗ$$

where ε = complex permittivity,$\;\varepsilon^{\prime }$ = permittivity, $\varepsilon^{\prime \prime }$ = dielectric loss, ω = angular frequency, and *σ* = electrical conductivity.

This review summarises all aspects related to the MW-assisted synthesis of different MOFs within the past 10 years. Although there have been multiple reviews on MOF design tactics for advanced electrochemical energy storage devices [17] and on the synthesis and application of MOFs [18,19], these reviews have discussed MOFs synthesised by different methods, including MW. We have also found few reviews on the MW-assisted synthesis of MOFs [20,21], their environmental advantages [22], and their biomedical applications [23]. The reviews discussing the MW-assisted synthesis of MOFs are not recent. Hence, we have included the recent modified reports of the MW-assisted synthesis and conditions of MOFs in this review. We have identified that there is a gap in reviews discussing the properties and advantages of MOFs synthesised under MW as compared with those synthesised under conventional synthesis. Although the biomedical applications of MOFs synthesised under MW irradiation have been exclusively reviewed [23], we have discussed their applications in diverse fields. We present in this review the properties of MOFs synthesised under MW and their recent applications, along with a critical evaluation of their future perspectives.

## 2. Properties of MOFs

The different properties of MOFs, such as stability, conductivity, porosity, and efficacy, are highly improved in MW-synthesised MOFs as compared with those synthesised by usual solvothermal or hydrothermal synthesis.

### 2.1. MOF Stability

The factors affecting the stability of MOFs are attributed to the coordination geometry of the metal and ligand, the environment under which they perform, the type of organic ligands and metal ions, the hydrophobic properties of the surface of pores, etc. [24,25]. The low stability of MOFs is due mainly to labile coordination bonds that bind the framework [26]. There should be strong coordination bonds within a stable MOF to withstand steric hindrance at metal nodes during the attack by guest molecules. The thermal stability of the MW-assisted synthesis of Ca-MOF, Sr-MOF [27], and rod-like aluminium terephthalate [MIL-53(Al)] [28] crystals increased above 450 °C with a high rate of carbon dioxide adsorption at room temperature. Their thermodynamic stability is analysed by the strength of the metal–ligand bonds of the framework. The strength of the metal–ligand bonds is correlated to the charge density because it is negatively related to the radius of the ion and positively to cation charges for a specific ligand. According to the hard–soft acid–base (HSAB) principle, metal ions possessing high charge densities and valencies form strong metal–ligand bonds, leading to the formation of a stable MOF framework [29]. Henceforth, hard base ligands (carboxylate anions) react with metal ions with a high valency (Zr^4+^, Ti^4+^, Cr^3+^) to form stable MOFs. The material Instiut Lavoisier (MIL) series was named for MOFs synthesised using metals of a higher valency (Cr^3+^, Al^3+^, Fe^3+^) to form MIL-101 [30], MIL-100, and MIL-53 [31], which was followed by the synthesis of Zr^4+^-based MOFs [32]. Soft metals with a lower valency (Cu^2+^, Mn^2+^, Zn^2+^, Ag^+^) react with soft azolate ligands to form stable MOFs based on the HSAB principle with the zeolitic imidazolate framework (ZIF) [33].

The stability of MOFs is also affected by kinetic factors such as the flexibility and rigidity of linkers. The rigidity can be imparted to the structure of a MOF by designing rigid linkers to control the conformation [34]. The presence of rigid organic linkers, metal clusters with a high valency and coordination numbers, and strongly bonded metal–oxo clusters contributes to the stability of MOFs. MW synthesis is used for the facile introduction of suitable linkers [35,36]. The approach of water as a guest molecule reduces the stability and can be hindered by the synthesis of hydrophobic surfaces [37]; the stability of MOFs can also be enhanced by hydrophobic coating [38]. However, MOF stability is limited by exposure to moisture and air, which is a long-standing problem for industrial applications [39]. The (Na,Cd)-MOF stored at ambient temperature for 3 months in 95% humidity exhibited high stability [40].

An efficient transfer of heat by dielectric heating occurs during MW-assisted synthesis. This heat transfer effectively occurs depending on the capability of the solvent to absorb microwave energy. Thus, a dielectric polar solvent molecule is required for the process. Polar solvents with an –OH group are favourable: water, ethanol, and DMF are the most suitable solvents. Nonpolar solvents are unable to absorb MW energy. Hence, ionic or polar additives should be introduced as additives if the reaction medium is less polar or nonpolar. A rapid transfer of energy takes place between the polar additive and nonpolar solvent, making a variety of chemical transformations feasible.

### 2.2. Porosity

MOFs should possess a high surface area and porosity for chemical and physical applications [41]. Proper linkers and metal nodes dictate the topology and size of pores in MOFs. Frameworks constituting a similar structural topology are termed “isoreticular MOFs”. Isoreticular MOFs can be designed using organic linkers with different functional groups and lengths, which aids in the analysis of chemical and structural factors that influence electrochemical processes [42]. Channels within the framework can be designed through a combination of mesopores and micropores, which further influences molecular diffusion. Additionally, a higher surface area improves the catalytic activity of MOFs. The coordination between the functional groups of organic linkers and metal nodes enhances the connectivity within MOFs, thus creating pores within crystallites. These pores can be accessed from the external environment by substrates allowing the mass to move in and out of the MOF structure. These pores can easily adsorb different gases and can be used for the removal of toxic gases from the environment. MW heating affects the size of pores in MOFs. The MW-assisted synthesis of MOF-74 (Ni) demonstrated that the capacity of the MOF to capture CO_2_ was enhanced on the elevation of the activation temperature from 150 °C to 250 °C [43]. This indicated that activated crystals at a higher temperature imparted open-metal Ni^2+^ sites and a higher amount of micropores in the MOF. A higher temperature plays a role in the fabrication of inner pores.

### 2.3. Crystallinity

The degree of the structural order within the product is termed “crystallinity”, which depends on the size of crystallites and lattice defects. The peaks in the diffraction pattern might become broad, depending on the angle of diffraction (q), thanks to lattice defects and the size of crystallites. The analysis of relative crystallinity can be conducted by including the full width half maximum (FWHM) of a particular peak. The unaffected peak with the highest intensity, due to the overlapping of other reflections, is generally considered. The same instrument should be used for the analysis of each sample to prevent instrumental errors. The modulation of the power of MW controlled the morphology and range of the particle size during the synthesis of MIL-53 (Al) [44]. The size of crystal particles became narrow with an increase in the absorption of MW power, as shown in Table 1. Additionally, the morphology of particles also varied with power. The MIL-53 formed a mixture of 50% of well-formed crystals and 50% of crystal-like structures with irregular shapes when the absorbed power was 269 W. In contrast, the morphology of the MOF was around 90% under the absorbed power of 1750 W. This was due to the different mechanisms of growth of the MOF, which depended on the reaction kinetics and temperatures [45].

### 2.4. Photoluminescence

The photoluminescent properties of MOFs have found several applications in the field of fluorescent sensors, biomedical imaging, photocatalysts, and nonlinear optics. The geometry of the phosphonic acid group is tetrahedral and interacts strongly with metal centres. The stability of the C-PO_3_ bond is high even at elevated temperatures. Thus, the presence of C-PO_3_ in MOFs imparts mechanical and thermal stabilities to the structure. The synthesis of [Ln_4_(H_6_btp)_2_(H_4_-btp)_2_(H_8_btp)(H_2_O)_16_]·12H_2_O [where Ln^3+^ = Ln^3+^, (La^0.9^ Eu^0.1^)^3+^ and (La^0.9^ Tb^0.1^)^3+^], having four phosphonic acid groups as organic linkers, was conducted using MW at 100 °C for 1 h [46]. Topological features were enhanced thanks to the presence of two aromatic rings and chelating moieties composed of phosphonic acid in H_8_btp. The photoluminescent and catalytic properties of the MOF motif were also augmented by these two groups [47]. The crystals of the Ln^3+^ MOF had a tetragonal space group with a metal centre at the asymmetric group. The stacking of three 1D organic cylinders occurred like a brick wall, and the free space between cylinders was occupied by water molecules. The cylinders comprised five organic linkers and eight metal centres in a zig-zag fashion. The stacking of organic linkers was parallel to each other and surrounded by metals. Thus, a 2,4,4,4-connected tetranodal complex with a layered network was present in the MOF. The metal centre was within the organic matrix. The π–π interaction aided in securely stacking the organic matrix. A large number of water molecules were trapped inside the channels and pores of the framework. The presence of photo- luminescent centres in (La^0.9^ Eu^0.1^ and La^0.9^ Tb^0.1^)^3+^ exhibited the photoluminescence properties of MOFs. In this line, another photoluminescent MOF, DUT-52, with high crystallinity was synthesised using MW within 25 min at 115 °C with terephthalic acid as a linker and Zn salt [48,49,50]. Optical properties were due to aromatic groups present in the molecule exhibiting photoluminescence at a wavelength higher than 287 nm. A highly intense emission was obtained with high excitation energy. The phenomenon of luminescence was due to the π–π* transition of the linker, along with electronic coupling between the cluster of [Zr_6_O_4_(OH)_4_]^12+^ and 2,6-naphthalene dicarboxylate (NDC) linkers.

## 3. The Analysis of the Efficiency of the Process

A procedure for synthesis is optimised by experimenting several times to identify the parameters under which the maximum output is obtained. The results of each experiment are compared to analyse the efficiency of the process under the different parameters: mass productivity and mass efficiency based on volume and time, quality of product, energy efficiency, and crystallinity.

### 3.1. Reaction Mass Efficiency (RME)

The measure of the yield of the reaction carried out in the stoichiometric amounts of reagents by factoring in all compounds participating in the reaction is termed “reaction mass efficiency (*RME*)”, which is analysed using Equation (2):

(2) $$RME=\frac{m_{p}}{m_{r1}+m_{r2}}\times 100$$

where *m_p_* = mass of product and *m_r_*_1_, *m_r_*_2_ = mass of reactants.

### 3.2. Energy Supplied (μ)

The mass of the product generated against the per unit measurement of the supplied energy is obtained from Equation (3):

(3) $$\mu =\frac{m_{p}}{E}$$

where *μ* = energy supplied to the system and *E* = total energy.

An appropriate determination of the heating mechanism of MOFs by enhancing the power of MW while keeping the total amount of absorbed energy fixed increased the rate of synthesis and improved the yield of MIL-53 (Al) [44]. The yield of the MOF increased from 15.9% to 36.8% on enhancing the absorbed power from 269 W to 682 W under a constant average absorbed energy of 51 kJ mol^−1^. This phenomenon established the fact that a selective heating mechanism occurs on the modulation of absorbed power while keeping the absorbed energy constant [51]. The rate of the reaction enhanced thanks to the generation of localised heating resulting from the short time of the reaction under high power. Thus, heat loss due to dissipation was less than that of the rate of heating, which improved the reaction rate [52].

### 3.3. The Space–Time Yield

The mass of the product derived from the reaction mixture per unit volume daily is termed “space–time yield *(STY*)”, which is calculated using Equation (4):

(4) $$STY=\frac{m_{p}}{V\times t}$$

where *V* = volume of the reaction mixture and *t* = time.

### 3.4. Modulators

The presence of chemical modulators is significant in controlling the growth and nucleation of MOFs [53]. Organic acids, such as monocarboxylic acids, benzoic acids, etc., are generally used as modulators in the synthesis of MOFs because acids have the capability to coordinate with metal ions by competing with polytopic organic linkers. The interaction of the modulator with metal ions leads to an enhancement of energy required to cross the nucleation threshold energy, aiding in diffusional growth [54]. These compounds positively impact the growth and nucleation of MOF particles over long reaction times. Additionally, monodispersed particles are also enhanced in the presence of modulators [55]. Modulators improve the morphology of the crystal structure and enhance catalytic and sensing capability [56]. Crystal sizes under MW heating decrease after the introduction of capping ligands as modulators [57]. Several capping agents were selected as modulators for curcumin, a natural product, to form medi-MOF-1. MOF crystals obtained by hydrothermal synthesis were spindle shaped in the absence of modulators, and the sizes of the crystals were reduced under MW heating. The introduction of capping agents highly affected the size and morphology of crystals; the morphology of spindle-shaped crystals was transformed into a cubic shape with the reduced sizes of particles. The growth of crystals is modulated by a competition in the rate of coordination between curcumin and the capping ligand. Crystal sizes were not affected even after the reaction time was prolonged in the presence of the modulators under MW heating. The presence of modulators can further improve the porosity of MOFs. The porous structure of medi-MOF synthesised under MW heating exhibited a smooth surface in the absence of modulators, which was transformed to form a hierarchical structure in the presence of modulators [50]. The modulators interact with the metal site and obstruct the growth of the crystal, leading to the generation of a hierarchical porous structure.

### 3.5. Kinetic Studies

**Figure 1:**
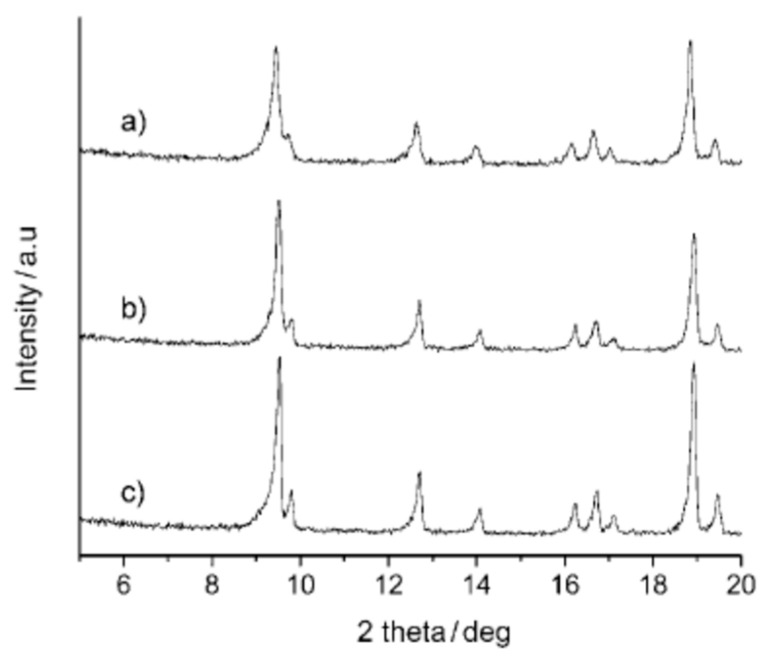
The XRD patterns of MIL-53 (Fe) synthesised at 70 °C by (**a**). US (35 min); (**b**). MW (2 h); and (**c**). CE (3 d). Reproduced with permission from [59].

MIL-53 [58] constituting of iron and 1,4-benezenedicarboxylate (BDC) or terephthalate was synthesised by three techniques: MW, ultrasound (US), and conventional electric (CE) heating for a comparative kinetic study [59]. The study revealed that crystallinity depends on the time, process, and reaction temperature, as shown in Figure 1.

The morphology of MOFs obtained through MW and US is homogeneous in that the crystallised phase is pure thanks to the formation of small MIL-53 (Fe) crystals as compared with that obtained by CE, according to the rate of crystal growth and nucleation. The rate of nucleation and the rate of reaction was the highest under US and the lowest under CE, while MW heating has an intermediate rate. The order was reversed for two parameters: activation energy (Ea) and the pre-exponential factor (A). The synthesis time was shorter under MW and US as compared with that under CE heating. The shorter synthesis time under MW was attributed to various factors: uniform heating, the alteration of interaction between the species, the generation of hot spots, the superheating of the reaction mixture, the augmentation of the dissolution of the reaction mixture, and the rapid heating process [60]. The properties of isostructural MOFs with similar ligands but different metals are ideal for studying the various properties of MOFs. Three isostructural CPO-27 [M_2_ (DHTP) (H_2_O)_2_·8H_2_O; M is Co [33], Zn [34], Ni [35], DHTP: 2,5-dihydroxyterephthalate] were synthesised using MW, US, and CE heating, and the properties of crystals were characterised [61]. The kinetics of synthesis indicated that US gave the highest rate, while CE gave the lowest rate. MW imparted an intermediate rate of synthesis, which was studied by dividing the rates into crystal growth and nucleation. The accelerated rate of the synthesis of CPO-27 under MW was based on the transient temperature and hot spots. The physical properties, such as surface tension, vapour pressure, and viscosity, rather than the chemical properties, such as basic, acidic, or the polarity of the compound, dictate the activation procedure [62]. CPO-27 synthesised by US had the smallest size and maximum porosity compared with that synthesised by the other two methods. The size of the compound obtained by MW was intermediate between US and CE, indicating that the sizes of the crystals are controlled by the rate of the crystal growth and nucleation [63]. An increase in supersaturation exponentially increases the rate of nucleation, leading to the formation of small particles thanks to the faster reaction rate and enhanced concentration of nuclei under MW [64].

Turbidity measurement is a technique to determine the pattern of crystallisation [65]. The transmission of light through the solution decreases as crystals are formed. The nucleation of MIL-53 (Al) was studied by this method [66]. The rapid synthesis of UiO-66 by MW was studied using a turbidity measurement, by differentiating reaction parameters for gaining an insight into the crystallisation process [67]. DMF was used as a solvent that decomposed into basic dimethylamine, aiding in the deprotonation of the organic linker to boost the formation of an assembly of inorganic clusters. Thus, the nucleation rate is augmented by enhancing the reaction temperature [68]. The onset of induction time distribution also increased with the reaction temperature. A long induction time was observed with solutions having higher concentrations owing to a high release of HCl, which prevented carboxylate linker deprotonation and slowed the assembly. The rate of nucleation was similar. Tetragonal ZnO_2_ nanoparticles were nucleated to form hexanuclear building units by the addition of water [69]. However, the formation of MIL-140A occurred at a high temperature in the absence of water [70]. Thus, it was established that UiO-66 is kinetically stable, while MIL-140A is thermodynamically stable. The nucleation of UiO-66 was prevented by a decrease in water. Benzoic acid as a modulator enhanced the onset of nucleation, and the modulator competes with Zr sites. The MOF was synthesised under MW, keeping reaction parameters similar to that of the turbidity process. Crystals were obtained within 5 min under MW with low crystallinity. However, highly crystalline compounds were obtained by the CE process. Nonpolar amorphous materials were obtained within 1 min with a low surface area. Nevertheless, the thermal stability of UiO-66 crystals in the presence of water and modulators was very high. The particle sizes of the crystals were not affected by the tuning of the synthesis time. Crystals were not formed in the presence of HCl as modulators under MW, while MIL-140A was synthesised in the presence of acetic acid as modulators under MW. Thus, kinetically and thermodynamically controlled products can be synthesised through a fusion of MW and modulation.

### 3.6. Activation of MOFs by MW

**Figure 2:**
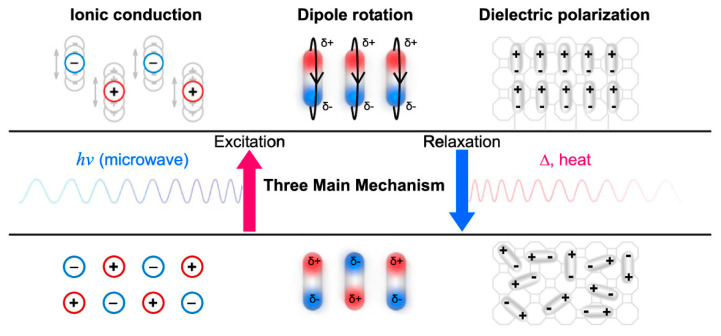
The mechanisms of the interaction of MW with the MOF. Reprinted (adapted) with permission from [75]. Copyright 2019, American Chemical Society.

MOFs are used in various applications, such as sensors [71], catalysts [72], drug delivery [73], and molecular separators. Solvents present in the pores of MOFs, along with solvents that coordinate with open-metal sites, should be eliminated before their applications in different spheres. This elimination process is termed “MOF activation”, which helps the guest molecule to freely move and appropriately interact with the MOF framework. The MOF is thermally activated at high temperatures under a vacuum for the evaporation of trapped solvent molecules and the dissociation of the coordinating solvent [74]. However, the structural integrity of the MOF is hindered because of the application of high temperatures and the transfer of heat energy by thermal diffusion into the MOF. MW activation has been effectively used for the removal of trapped molecules. Heat energy is propagated by convection or thermal diffusion thanks to the molecular collision or vibration of the lattice, while heat energy moves into the centre of a chemical compound directly in the form of a wave through MW irradiation. Electrons present in the MOF couple with the electromagnetic field of MW for transferring MW energy at the molecular or atomic level. The three mechanisms of MW activation are the dielectric polarisation, ionic conduction, and dipolar rotation of field-induced polar molecules or dipolar molecules, as shown in Figure 2.

Charged particles move in a translational oscillation motion on being excited by MW. Dielectric solids and dipoles present in polar molecules are excited through oscillation polarisation and rotational oscillations. They emit heat energy while relaxing to the ground state. This heat energy is utilised for the elimination of trapped and coordinating solvents from the MOF framework. HKUST-1 was prepared by solvothermal synthesis, and the effect of MW activation was studied [75]. The result was analysed on the basis of the dissipation factor, as shown by Equation (5):

(5) $$tan\delta =\frac{\varepsilon^{\prime \prime }}{\varepsilon^{\prime }}$$

where tanδ = dissipation factor,$\;\varepsilon^{\prime }$ = dielectric constant, and$\;\varepsilon^{\prime \prime }$ = dielectric loss.

The dissipation factor is a parameter for measuring the capacity of a material at a particular temperature and frequency for the conversion of electromagnetic energy into heat. A higher dissipation factor indicates rapid heating and effective absorption. There are three types of organic solvents based on MW absorption capability, as shown below:

a. The value of tanδ more than 0.5 indicates high MW-absorbing solvents (EtOH, DMSO, and ethylene glycol)
b. The value of tanδ within 0.1–0.5 indicates medium MW-absorbing solvents (water and CH_3_CN)
c. The value of tanδ less than 0.1 indicates low MW-absorbing solvents.

The efficiency of the conversion of MW energy into heat energy is directly dependent on the value of *tanδ*. This value is lower for acetonitrile and DMF as compared with that of MeOH and EtOH. Different parameters contribute to the activation time: coordination strength, the dissociation energy of the coordinating solvent, the dissipation factor, and the energy of solvent evaporation. The dissipation factors of solvents increase from MeCN, DMF, and MeOH to EtOH, while the strength of coordination solvents (MeOH, MeCN, and EtOH) is similar. Conversely, DMF as a solvent has a much higher value [76]. Thus, HKUST-1 was effectively activated with intact structural integrity depending on the parameters of the different solvents. Among the four solvents, MeOH was found to be a suitable solvent for MW activation within 4 min thanks to its high dissipation factor, low boiling point, and coordination strength for HKUST-1. UiO-66 and MOF-74 required 3 min and 5 min, respectively, in MeOH, which justifies the conclusion that MeOH is a suitable solvent of MOFs for MW irradiation.

### 3.7. The Absorption of MW

The absorption pattern of MW by Fe-MOF was explicitly studied by analysing the maximum reflection loss due to absorption by the MOF with a particular thickness [77]. The capability of MW absorption is influenced by various properties: magnetic permeability, skin depth, nonzero magnetic susceptibility, conductivity, and dielectric permittivity. The size distribution was large with irregular shapes of Fe-MOF particles. The optimal thickness of the MOF for MW absorption was found to be 2.50 mm. The thickness of the MOF controlled the frequency of the reflection loss peak (f_peak_); the f_peak_ value moves towards the lower frequency from the higher frequency with the increasing value of d. Fe-MOF stores a huge amount of energy at a low frequency, which vanishes with the increase in frequency. The conductivity of the MOF is high within the range of microwave frequency. An increase in frequency increases the microwave conductivity with a decrease in the skip depth. The reflection loss peak value (RL_peak value_) is augmented by the magnetism of Fe-MOF, which in turn enhances the efficiency of MW absorption. MW absorption is influenced by electrical relaxation and not by magnetic relaxation. Intrinsic MW absorption depends on the rotation of polar groups. Because Fe-MOF has a symmetrical geometry, the polarisation of the molecule is neutralised within the molecule. The incident electric field that is due to MW is induced thanks to the rotation of polar arms within the motif. There is also inherent polarisation thanks to structural defects’ initiating MW absorption. Unpaired electrons in metal ion centres also contribute to magnetic resonance’s aggravating the resonance of MW radiation with spin states. Thus, Fe-MOF exhibited effective MW absorption. A MOF consisting of cobalt CPT-1-Co[Co_2_O-(cptpy)2(DMF)] (Hcptpy = 4′-(4-carboxyphenyl)-4,2′:6′,4′′-terpyridine, DMF = N,N-dimethylformamide) was synthesised by the solvothermal process and pyrolised at 700 °C to obtain a Co/C-700 composite [78]. CPT-1-Co is a 3D framework where each dinuclear Co^2+^ cluster is linked to two carboxylate anions present in organic ligands, two oxygen atoms of DMF, and four nitrogen atoms present in cptpy anions. Each cluster has two Co^2+^ ions linked through a μ_2_–O bridge. The clusters and organic ligands form three types of cages shared through either the vertex or the edges. The structure consists of intercrossing 3D cavities that add porosity. Co/C-700 exhibited ferromagnetic behaviour. The two parameters, magnetic loss tangent (tan *δμ* = *μ*″/*μ*′) and dielectric loss tangent (tan *δε* = ε″/ε′), were used for analysing the absorption of MW. These parameters were the same for Co/C-700 at a lower frequency range of 2–12 GHz, which enhanced MW absorption. It was concluded that the value of the reflection coefficient should be smaller than −10 dB for 90% MW absorption by the nanomaterial of 1–5 mm thickness. Thus, better MW absorption occurs within a low-frequency range.

## 4. The MW-Assisted Synthesis of MOFs

**Figure 3:**
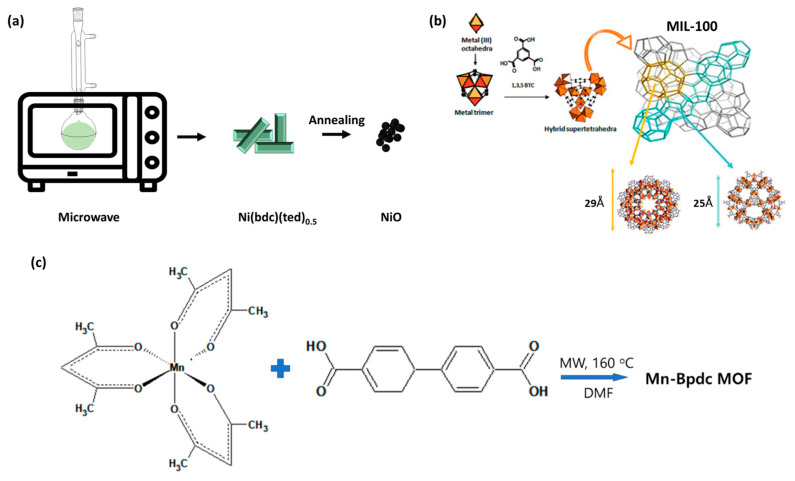
(**a**) MW-assisted synthesis of NiO nanoparticles, (**b**) the formation of thin films by MIL-100, and (**c**) Mn-MOF [79].

The synthesis of compounds using MW heating is widely used in the field of synthetic organic and inorganic chemistry, as shown in Figure 3 [79]. The major asset of using MW irradiation is that the whole sample gets uniformly heated, and the process is independent of the requirement to transfer heat within the mixture [80]. Because the volume of the reaction mixture is independent of the heating process, the scaling-up procedure is less complicated.

Cr-MIL-100 was the first MOF to be synthesised under MW by heating at 220 °C for 4 h [81]. The method was later modified by reducing the time to 1 h, which furnished the MOF with similar textural and physicochemical properties. An efficient zirconium-based MOF (UiO-66) was synthesised by MW by using acetic acid as the additive and trifluoroacetic acid as the modulator for creating catalytic metal sites after removing them from the metal clusters [82]. However, amorphous products were formed under low reagent conditions. Alternately, crystalline products were obtained by enhancing the reagents in the presence of modulators. Low-quality crystals formed in the absence of modulators. Modulators also enhanced the absorption capacity of MW and maintained the required temperature. Crystals were free of metal defects. In another study, a crystalline Zr-MOF was synthesised by using MW with highly defined crystals with an octahedral shape possessing sharp edges [83]. The hydrogen storage capacity was less than that synthesised by CE heating, according to the smaller pore volume and smaller surface area of the MOF synthesised by MW. MOF-199 or HKUST-1([Cu_3_(BTC)_2_] [BTC = 1,3,5-benzenetricarbox-ylate]) has a significant structure based on its high chemical stability and pore volume. It was synthesised by using MW within 30 min with a yield of 77% [84]. The crystal size was increased by enhancing the time of crystallisation on the basis of Ostwald ripening, according to which large homogeneous crystals were formed from the smaller octahedron units of crystals. An equilibrium known as a crystallised–dissolved equilibrium state was obtained, where tiny particles remained mixed with larger particles [85]. The hydration of MW-synthesised MOF-199 was less compared with that from the solvothermal synthesis. The agglomeration decreases under MW and boosts the synthesis of crystals with a high surface area. Several MOFs were synthesised by fast and facile methods from transition metals (Cu, Co, Ni, and Fe) using MW to produce different coloured crystals [86]. The crystallinity of Cu-BTC was superior, while the crystallinity of Co-BTC was lower owing to impurities from BTC than those synthesised by the CE method. Ni-BTC exhibited a flower-like morphology. The crystals of all MOFs were small and homogeneous with good surface areas. Mg(BTC) [Mg(H_1.5_btc)_2/3_(btc)_1/3_(DMA)_2_.(DMA)_1/3_] was prepared by using MW under solvothermal conditions within 1 h [87]. The size of small cylindrical crystals was smaller with more thermal stability with an increase in temperature. The most effective crystal was obtained at 220 °C, and it was partially soluble in water.

### 4.1. The Solvent-Free Condition

The synthesis of MOFs by MW requires the absorption of electromagnetic waves by metal ions, especially under solvent-free conditions. Heating spots are generally present in organic solvents when the MW reaction is carried out in an organic solvent. Among different metal ions, cobalt is an effective ion in that the spots get superheated within the metal by MW. This activates the formation of the complex between ligands and metal ions to form microporous 3D MOF structures. Zeolitic imidazolate frameworks (ZIFs) are zeolites with topologically isomorphic structures. The transition metal ions in ZIFs are coordinated tetrahedrally and connected through imidazole linkers [88]. They are stable at very high temperatures; hence, they can be used at elevated temperatures [33,89]. A ZIF was synthesised using cobalt (ZIF-67) under MW under solvent-free conditions [90]. Reactants were milled by hand to enhance the absorption of moisture prior to MW treatment. The synthesised ZIF-67 was crystalline with rhombic dodecahedral crystals of 500 nm in size and was smaller than crystals obtained by conventional methods. The reduced size was attributed to the fast rate of growth and the nucleation process. The MW treatment, however, reduced the size of the pores. The solvent-free treatment enhanced the surface area of the crystals and prevented reagent poisoning. Additionally, the reduction of heating time augments the volume of micropores and the surface area, to a considerable extent. This report proved that cobalt is an essential metal for the synthesis of MOFs under solvent-free conditions.

### 4.2. Continuous-Flow MW-Assisted Reactors (CFMR)

**Figure 4:**
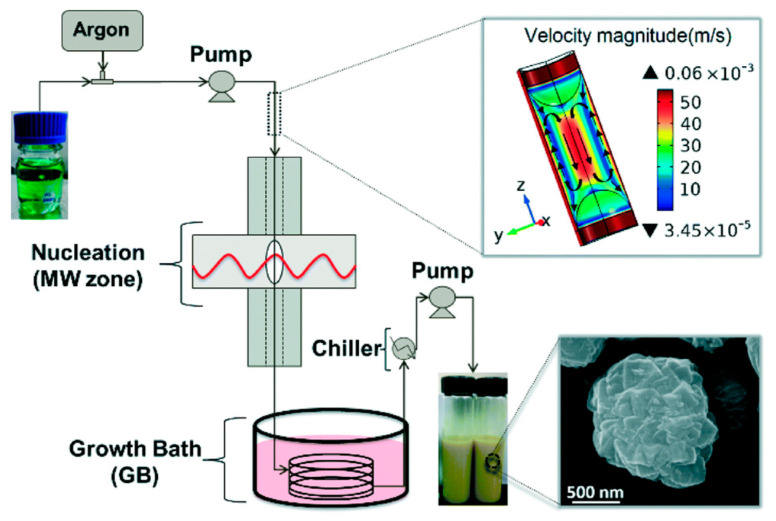
The schematic representation of the CFMR for the synthesis of MOF-74 (Ni). Reproduced with permission from [91].

A CFMR was used for the synthesis of MOF-74 (Ni) by applying flow through a gas–liquid segment for controlling the particle size through a fast nucleation process [91]. The procedure was controlled by manipulating the temperature of the nucleating zone in the MW reactor (T_MW_) and the growth zone in the heating bath for the synthesis of highly crystalline MOFs, as shown in Figure 4.

The reaction time was prolonged, the heat transfer was uniform, and the fouling of the reactor walls was minimised by the application of gas–liquid segmented flow [92]. Particles deposited because of fouling caused clogging and also created hot spots of MW. The generation of crystalline materials increased with the increase of the nucleation temperature. Agglomeration was also caused by the high absorption of MW by the small grains. The asset of this technique is that intracrystalline diffusion can be prevented thanks to agglomeration, which separates the boundaries between grains and decreases the surface area for adsorption. The CFMR demonstrated an improvement in the reaction condition by shortening the reaction time and the better utilisation of reagents [92,93]. MOF-74 (Ni) was synthesised by using the CFMR in the presence of benzoic acid (BA) as a modulator [94]. The reaction nucleation rate was decreased by enhancing the concentration ratios of BA to Ni^2+^ ions. There occurs a competition between BA and the polytopic organic linker for the Ni^2+^ cation. The nucleation threshold is affected by the interaction between the modulator and the metal ion, thereby decreasing the growth rate. The effective diameters of particles increase, thus decreasing the distribution of the particle size. However, the product formed was amorphous at a very high ratio of BA to Ni^2+^. The nucleation process could be separated from the growth process by heating the reagents in a heating bath after MW irradiation. MOF-74(Ni) was used for the preparation of composite membranes with Nafion to form MOF-74(Ni)/Nafion, which was used for effectively removing Ag metal. Ag clusters were formed on the MOF owing to the reduction of Ag^+^ by n-propanol trapped within the membrane. A lower amount of Cu^2+^ and Pb^2+^ was also removed from the wastewater. Another study revealed the large-scale synthesis of three MOFs (UiO-66, MIL-53(Al), and HKUST-1) by using the CFMR [95]. The three MOFs were prepared under different reaction conditions using DMF as the common solvent. UiO-66 was obtained in a 94% yield within 7 min at 280 W under atmospheric pressure in the presence of acetic acid as the modulator, and water was used to form zirconium oxoclusters for the secondary binding of the MOF. The reaction was carried out with reagents in the stoichiometric amount. The reaction for the formation of MIL-53(Al) was carried out at a pressure of 3 bar and at 240 W for 4 min with a yield of 65%. The formation of inorganic building units, along with the yield, was promoted by the presence of water. The metal and ligand were mixed in a stoichiometric amount to synthesise HKUST-1 at 4 bar of pressure and 360 W for 1 min with a yield of 96%. All three syntheses were scaled up for a larger reaction process to be effectively applied in industries.

### 4.3. MW-Assisted Dry-Gel Conversion (MW–DGC)

**Figure 5:**
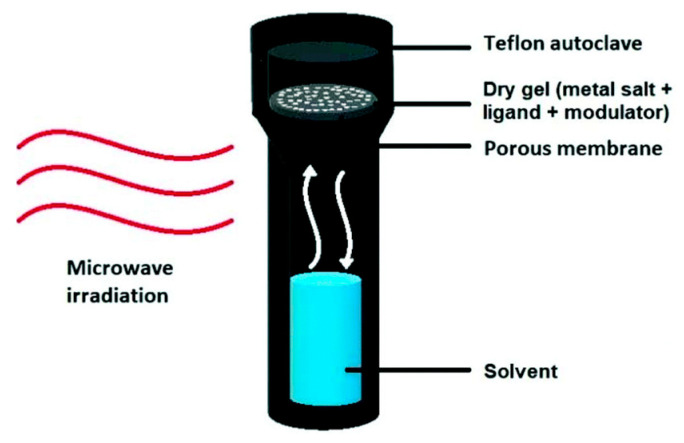
The schematic representation of MW–DSC. Reproduced with permission from [98].

The MW–DGC apparatus consists of a porous plate placed on top of an autoclave container. The reagents are placed on the surface of the porous plate, while the solvent is placed at the bottom of the container, as shown in Figure 5 [96]. The solvent can be recovered and reused by this process. Moreover, the reactor size is also small [97].

Four MOFs (UiO-66, Fe-MIL-100 (Basolite F300), aluminium fumarate (Alfum, Basolite A520), and MIL-140A) were synthesised by MW–DSC to study their properties [98]. Fe-MIL-100 was synthesised in water in the absence of modulators within 3 h with a small BET surface area, porosity, and crystallinity as compared with the CE–DGC method thanks to the formation of a semiamorphous structure in water [99]. UiO-66 was obtained within 1 h at 180 °C in the presence of HCl and BA as modulators, while MIL-140A was obtained within 1.5 h at 160 °C in the presence of BA as the modulator with acceptable parameters for crystallinity. The dry gel was formed with a thick consistency thanks to BA, while the surface area was increased thanks to HCl. However, HCl decomposed DMF into formic acid and dimethylamine, hindering the reuse of the solvent. Hence, the process was modified by using ionic liquids as a substitute for HCl. The crystal sizes of UiO-66-HCl (100–200 nm) and MIL-140A (50–250 nm) obtained in ionic liquids were uniform but were agglomerated. Alfum was also mesoporous thanks to the presence of defects. Solvents used for the synthesis were reused thrice in the MW–DGC process.

### 4.4. The MW-Assisted Ball-Mill Process

The ball-milling process of solids and liquids under MW changes the conventional route of the reaction and also augments the reaction speed. Mechanical energy is transferred to the reaction mixture by the ball-milling process. Four MOFs were synthesised by using terephthalic acid, trimesic acid, Co(CH_3_COO)_2_·4H_2_O, and Cu(CH_3_COO)_2_H_2_O [100]: MOF-1 [H_3_BTC, Cu(OAc)_2_·H_2_O], MOF-2 [H_3_BTC, Co(OAc)_2_·4H_2_O], MOF-3 [H_2_BDC, Cu(OAc)_2_·H_2_O], and MOF-4 [H_2_BDC, Co(OAc)_2_·4H_2_O]. Congo red is a dye that remains positively charged in water and is chemically and physically adsorbed by MOFs. There occurs a π–π conjugation between π electrons in the benzene ring of MOFs and π electrons of C=C in Congo red. Hence, the adsorption of Congo red was studied by different MOFs. The adsorption rate was initially enhanced, which decreased with time. Absorption values were different depending on different organic ligands and metal ions.

## 5. Advantages of MW-Synthesised MOFs According to Their Geometry and Applications

### 5.1. Catalysts

**Scheme 1:**
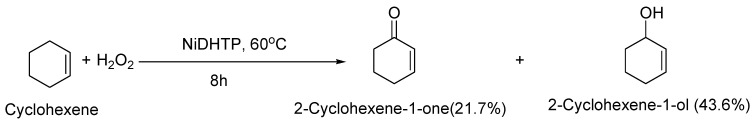
Oxidation of cyclohexene.

A porous MOF was designed using nickel(II) dihydroxyterephthalate (Ni-DHTP) under MW irradiation and used as a catalyst for the oxidation of cyclohexene in the presence of H_2_O_2_ [101]. Ni-DHTP was synthesised at different temperatures, which exhibited a highly crystalline structure. However, the crystals were larger at lower temperatures. The formation of smaller crystals, along with rapid nucleation, occurred as the temperature increased beyond 90 °C. This decreased the crystallinity of NI-DHTP. Additionally, the pore volume and surface area decreased at elevated temperatures because temperature played a pivotal role in the crystallisation process within a short duration. For the oxidation of cyclohexene, H_2_O_2_ (1:1) in the presence of Ni-DHTP as a catalyst at 60 °C formed 2-cyclohexene-1-ol (43.6%) with the highest selectivity, as well as forming 2-cyclohexenone, as shown in Scheme 1. The initial epoxidation step takes place through the interaction of cyclohexene with unsaturated Ni(II)-active sites. The catalyst did not leach at room temperature, while the reaction path was heterogeneous.

**Scheme 2:**
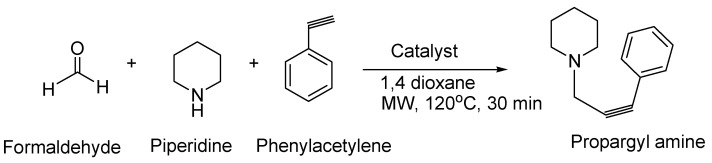
The one-pot synthesis of propargyl amine.

Au nanoparticles were embedded in MOF-199 [Cu3(1,3,5-trimesic acid)_2_(H_2_O)_3_] as a support to form Au/MOF-199 to catalyse coupling reactions [102]. Au nanoparticles were dispersed throughout the metal framework. DMF was used as a solvent because it also behaves as a mild reducing agent at high temperatures [103]. The hard C=O group of DMF interacts with the hard oxophilic cation (Cu^2+^), while the soft C–N group of DMF interacts with Au NPs on the basis of the HSAB principle facilitating the formation of the product. The formation of the MOF in pure DMF was fast because gold cations were easily reduced under MW and were dispersed on the outer surface of the MOF. This prevented Au clusters from aggregating. The nucleation of reactants was fast under MW thanks to uniform heating throughout the mixture and formed ultrafine Au NPs. The MOF was selectively used as a catalyst for the synthesis of propargyl amine in one pot under MW, as shown in Scheme 2.

**Scheme 3:**
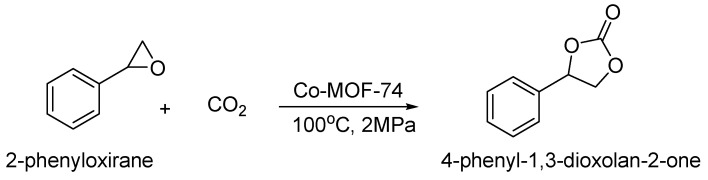
The cycloaddition reaction using Co-MOF-74.

The synthesised Co-MOF-74 was used as a catalyst for the cycloaddition reaction between styrene oxide and CO_2_ in chlorobenzene to furnish 4-phenyl-1,3-dioxolan-2-one, as shown in Scheme 3 [104]. The rate of conversion increased with the increase in temperature, while the same decreased with the decrease in the pressure of CO_2_ because the dissolution rate of the gas in chlorobenzene increased under elevated pressure. Thus, the MOF is a promising candidate for the cycloaddition reaction.

**Figure 6:**
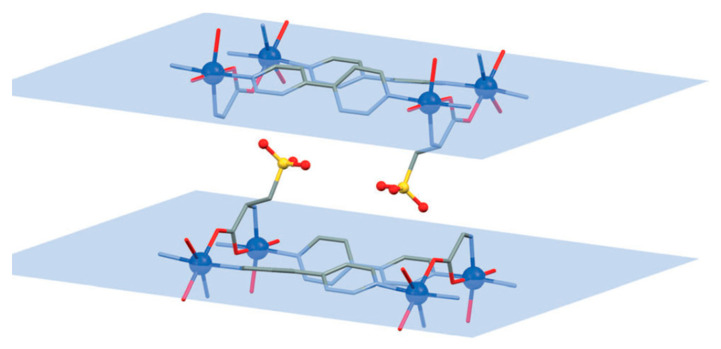
The mercury diagram showing 2D rectangular grids in 2D CCB. Reproduced with permission from [105].

In another attempt, a Co-MOF was functionalised with sulfonate groups under MW and effectively used as a catalyst for the synthesis of cyclic carbonates by the addition of CO_2_ [105]. A 2D cobalt–cysteate coordination polymer (2D-CCB) [{Co(4,4′-bipy)(l-cys)(H_2_O)}·H_2_O]_n_ was prepared within a short time (10 min) using a 1:1 mixture of water and methanol with a brick wall topology. Rectangular grids were stacked through hydrogen bonds, within which SO_3_ groups were freely oriented, as shown in Figure 6.

The MOF behaved as a multifunctional catalyst thanks to the presence of a sulfonate anion, a lone pair of electrons on nitrogen and oxygen atoms, and a cobalt metal centre. The lone electron pairs are basic sites of the MOF [106]. The acidic sites are equivalent to the number of cobalt centres. The MOF catalysed the Knoevanagel reaction, a base-catalysed reaction; it was effectively used for the synthesis of styrene carbonate from styrene oxide and carbon dioxide with 99.9% selectivity. The activation of the epoxide ring was initiated by the cobalt metal centre. The recycled catalyst was also valuable, with a minor loss in its efficiency owing to the minor loss of crystallinity.

**Scheme 4:**
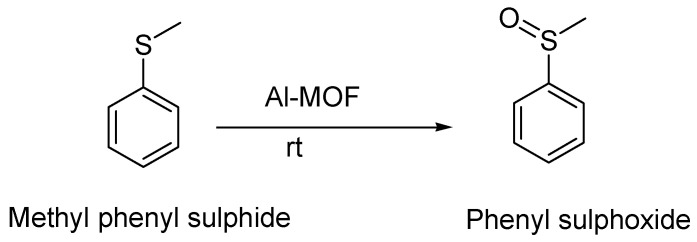
The oxidation of methyl phenyl sulphide to phenyl sulfoxide.

Another 3D porous MOF-205 [Zn_4_O(2,6-NDC)(BTB)_4/3_] was synthesised by using MW at 150 °C for 20 min, and the MOF was used as a catalyst for the cycloaddition reaction with CO_2_ as a substrate [107]. The cubic crystals of MOF-205 have their frameworks made of clusters of Zn_4_O bonded through ditopic and tritopic H_2_NDC and H_3_BTB, respectively, to form mesopores and micropores. The dodecahedral cages of mesopores were formed by eight units of BTB, [Zn_4_O]^6+^ units and four units of 2,6-naphthalenedicarboxylate, while the micropores were formed by clusters of Zn_4_O, four units of BTB, and two units of 2,6-naphthalenedicarboxylate moieties. The distance between the Zn–Zn bond was found to be similar to that between MOF-5 and MOF-177 [108]. The MOF was used to catalyse the synthesis of cyclic carbonates from epoxides and CO_2_ with 99% selectivity in the presence of tetrabutyl ammonium bromide (TBAB) as the solvent. The reaction did not proceed under the solvent-free condition, indicating the role of the solvent in the reaction. The interaction of the substrate and catalyst was enhanced thanks to the presence of micropores, while bulky substrates (epoxides) were adsorbed by the mesopores of the MOF. The adsorption of CO_2_ by MOF-205 was found to be very high according to its large pore volume and surface area. In another work, five MOFs with aluminium metal (NH_2_-MIL-53, MIL-53, MIL-100, DUT-4, DUT-5) were prepared by MW irradiation in ethanol or water within 2 h and at a temperature range of 120–210 °C [109]. The catalytic efficacy of MOFs was analysed for the conversion of methyl phenyl sulphide to phenyl sulfoxide, as shown in Scheme 4. The size of the MOF crystals synthesised under MW was smaller, with smaller surface areas than those prepared by CE. The structure of MOFs was stable and porous. The oxidation of methyl phenyl sulphide in the presence of H_2_O_2_ at room temperature was 99% selectivity by all catalysts. The yield of the product was increased with extended reaction time.

**Scheme 5:**
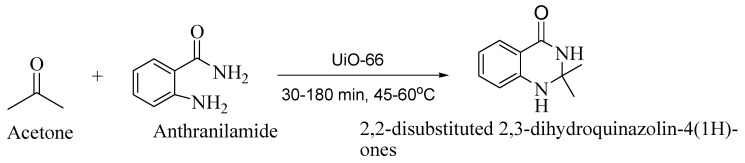
The cycloaddition reaction.

Linear 2,6-naphthalenedicarboxylic acid (H_2_NDC) linkers and trigonal prismatic Fe_3_O(–CO_2_)_6_ clusters with triangular 4,4′,4′′-benzene-1,3,5-triyl-tris(benzoic acid) (H_3_BTB) were used to synthesise MOF-97 at 120 °C in DMF for 24 h with acetic acid as the modulator [110]. Crystals were body-centred cubic with the shape of a distorted octahedral cage. The primary cage was built of six units of the trigonal prismatic clusters of Fe_3_O(–CO_2_)_6_. The complete structure was made by the combination of cuboctahedral cages to form 1D pore channels interconnected through the cubic lattice structure. MOFs having a single linker displayed minimal transitive nets with the highest frequency according to their symmetry. The ratio of the lengths of linkers was significant for acquiring the best symmetry of MOFs with two or more linkers. Tritopic and ditopic ligands were appropriately sized for MOF-907, indicating that the crystal was symmetric and highly porous. MOF-97 was used as a catalyst for the polymerisation of methyl acrylate under MW to form products with high yields. The catalytic activity of UiO-66 was tuned during its synthesis by MW and was examined as a catalyst for the solvent-free synthesis of 2,2-disubstituted 2,3-dihydroquinazolin-4(1*H*)-ones [111]. UiO-66 was synthesised within 3 min at 110 °C. The catalyst was used for the cycloaddition reaction of acetone and anthranilamide with 96% yield, as shown in Scheme 5. The catalyst was recycled five times and was reported to be effective and a promising catalyst for large-scale applications.

UiO-66-NH_2_ was prepared from Zr alkoxide and Zr oxychloride under MW irradiation at 120–180 °C [112]. The crystal has good texture and high crystallinity. It shows good photocatalytic activity in extracting sulfamethoxazole from water. The MW-assisted synthesis of NH2-MIL-125(Ti) MOFs was highly crystalline and porous; it was effectively photodegraded diclofenac [113]. The MOF was stable, without any loss of its photocatalytic activity. Zn-MOF was prepared by the MW-assisted ball-milling process that photocatalytically degraded Congo red and tertracycline [114].

MOF-derived ZnO composites were embedded with reduced graphene oxide (rGO) using MW. The degradation of methylene blue (MB) by the photocatalytic activity was studied by using the MOF [49]. Mixing between rGO and ZnO was highly homogeneous, as indicated by the densely populated ZnO particles on rGO nanosheets. The photocatalytic activity was promoted by different bridged ZnO particles through rGO sheets separating the photogenerated carriers. The composite possessed a similar structure of wurtzite as ZnO. The excitonic intensity of photoluminescence was reduced thanks to the presence of rGO in the composite. Thus, the recombination of holes and electrons from the photoinduction of ZnO could be prevented. The photocatalytic activity was also effectively improved thanks to the large surface area. However, the presence of an excess amount of rGO might create recombination centres and block the electron passage [50].

### 5.2. Adsorption: Gases and Organic Compounds

Environmental pollution has been a long-standing problem, and MOFs (MOF-177 and MIL-101) have demonstrated effective performance levels in the removal of CO_2_ under certain pressure conditions from the atmosphere [30,115]. MOFs functionalised with amino groups (MIL-53) and chabazite zeolites were reported to adsorb CO_2_ [116]. MOF-5 [Zn_4_O(BDC)_3_] was prepared by solvothermal synthesis under MW irradiation at 105 °C for 30 min to obtain cube-shaped and -structured crystals [117]. MOF-5 crystals obtained by CE heating were not as pure as those obtained by MW heating. The pores of the structure were well formed, and the reaction rate was faster thanks to the localised superheating effect of the MW process. The formation of MOF crystals with high Langmuir surface areas was also favoured under MW thanks to the nucleation of MOF growth sites. The MOF-5 was found to effectively adsorb 3.8wt% of CO_2_ under normal atmospheric pressure. Another report indicated the synthesis of MOF-5 [Zn_4_O(BDC)_3_] under MW at ambient pressure within 84 s at 80 °C, which exhibited similar properties to the MOF prepared by the general solvothermal process [118]. Co-MOF-74 synthesised using MW at 130 °C for 1 h was used for CO_2_ adsorption; its catalytic activity was explored for the cycloaddition of CO_2_ and styrene oxide to form cyclic peroxo compounds [104]. These MOF crystals had large surface areas and yields compared with those obtained by solvothermal synthesis. They had a single phase, with particles having the shape of hexagonal column structures. The MOF contained a long single type of pore with hexagonal channels of a single dimension. These channels adsorbed excess water via intermolecular attraction. The Lewis acidic sites in Co-MOF-74 are hydrophilic. The sites of oxygen present in the metal complex and organic ligands interact with water molecules through hydrogen bonding. The presence of the benzene ring in the structure makes it hydrophobic. MOFs effectively and selectively adsorb CO_2_ at open-metal sites, which are Lewis acids. MOF-74 (M = Ni, Mg) was synthesised using MW and examined for the adsorption of various gases [119]. The crystals of Ni-MOF-74 formed small polyhedral aggregates with a uniform size. The volume of micropores and the surface area were enhanced for the metal MOF-74. MOF-74 was also very porous and effective for the adsorption of carbon dioxide, nitrogen, methane, and other organic substrates. UiO-67 was prepared by MW in the presence of HCl and BA as modulators. UiO-67 was further used to adsorb methane and CO_2_ [120]. The dipole moments of the solvent molecule positively contribute towards the synthesis of MOFs. Hence, DMF possessing a large dipole moment was used as the solvent [121]. Modulators dictated the property and crystallisation of UiO-67. Here, 40 equivalents of BA were required to obtain excellent crystals, while the amorphous product was obtained with 10 equivalents of BA with the formation of zirconia gel [122]. Additionally, 185 equivalents of HCl were required for forming optimum octahedral-shaped crystals with well-defined edges and faces. The size of crystals increased with the increase in the number of modulators thanks to competition between modulators and linkers, which in turn decreased the number of nuclei. This phenomenon of linker deficiency increased the size of the crystals [123]. The surface area of the crystals obtained in the presence of BA was higher than that of HCl thanks to the presence of amorphous byproducts in the case of HCl. Crystallinity and related properties were improved compared with the conventional method of synthesis. The higher surface area of the MOF exhibited improved adsorption of CH_4_ and CO_2_ at 50 °C and 25 °C, respectively. The affinity for CO_2_ was more than that of CH_4_, which indicated that UiO-67 was selective for CO_2_.

MOF 235, an orange crystal with an octahedral shape, was obtained under MW within 15 min [124]. Larger crystals with a microporous structure and high yield were obtained on increasing the irradiation time [100,125]. The adsorbing capability of the MOF was affected by the distribution of the size of pores. The most effective adsorption occurred when the pore diameter of the adsorbent was 1.7-fold larger than that of the adsorbate [126]. The well-ordered structure of MOF 235 was lost, leading to an increase in the volume of micropores and the BET surface area after the dye was trapped within the pores. The octahedral crystals had iron trimers sharing the corners of the octahedron. Trimers were connected through the linear links of terephthalic acid. Each trimer had Fe-(μ3-O)-Fe forming an angle of 120°, and both Fe ions were separated by 3.33 Å [127]. The adsorption rate of acid chrome blue K by MOF 235 was studied and found to be proportional to the square of the number of unoccupied adsorption sites, indicating a pseudo-second-order rate model.

### 5.3. Drug Loading

Biocompatible drugs are encapsulated within MOFs for proper drug delivery. Amino acids, nucleotides, and peptides are considered biocompatible organic linkers that are connected to different metal ions (Zn, Ca, and Fe) in the MOF [128]. An interaction exists between the potassium ion and γ-cyclodextrin-MOF (γ-CD-MOF), possessing uniform channels and large spherical voids. A synthesis of γ-CD-MOF was reported using a pharmaceutical compound (PEG 20000) as the modulator under MW for a short time [129]. The crystallinity with cube shapes was improved under MW compared with under CE heating. However, crystallinity was hampered by prolonging the reaction time to 120 min [128]. The crystalline structure was independent of the reaction temperature, but the solvents had a significant role in dictating the shape of crystals. The irregular hexagonal crystals were converted to cubic crystals by enhancing the amount of methanol in the methanol–water solvent system. The nucleation of MOF crystals was triggered by methanol, which imparted stability to the growth of the crystal face. The sites for nucleation were reduced under the condition of low supersaturation [130]; crystals were not formed in a low amount of methanol, which initiated a low supersaturation state. The modulator aided in the formation of nanometre-sized crystals and also diminished the surface area of crystals. A nonsteroidal and weakly acidic anti-inflammatory drug (fenbufen) was used for examining its adsorption by γ-CD-MOF. The MOF with crystals in the range of 100–300 nm had the maximum adsorption capability. Adsorption occurred thanks to interaction through hydrogen bonding between the –COOH group in fenbufen with hydroxyl groups of dextrin in γ-CD-MOF.

In another drug-related study, two MOFs (Zr-UiO-66, Hf-UiO-66) were synthesised using MW for 3 min at 110 °C and were used for the in vitro adsorption of curcumin (CUR), a drug molecule [131]. The surface areas and pores of both MOFs were increased under MW compared with those under CE heating. The adsorption of CUR by the synthesised MOF was higher thanks to the large surface area, the small size of the particles, and the strong interaction between the carbonyl group of CUR and the metal clusters present in the MOFs [132]. Among the two MOFs, the stronger acidic character the the superior oxophilic character of Hf-oxo clusters enhanced the adsorption of Hf-MOF compared with Zr-MOF. The negative ΔG value indicated spontaneous adsorption. The reaction was exothermic. CUR was randomly adsorbed throughout the MOF despite the repulsion between CUR and the framework because the molecular weight of CUR is very large. Atoms surrounding CUR on the MOF are differently charged, indicating the attraction between CUR and the metal framework.

### 5.4. The Separation of Anthraquinones

Nano-MIL-101 (nMIL-101) was synthesised by MW heating and used for separating anthraquinones (emodin, rhein, aloeemodin, physcion, and chrysophanol) by using a pseudostationary phase (PSP) and dispersed particle extraction (DPE) in capillary electrophoresis (CE) from water samples [109]. Here, nMIL-101 was dependent on pH alteration within 7.5–9.5. Negative charges were enhanced on increasing pH values. The trimeric octahedral clusters of chromium and terephthalate ligands are the building units of nMIL-101. Further, nMIL-101 has mesoporous pores for adsorbing anthraquinones. Adsorption occurs thanks to various interactions: carbonyl sites in the unsaturated Cr metal within pores and the π electrons of aromatic rings, π–π bonding, hydrogen bonding between aromatic terephthalic rings in nMIL-101, and the aromatic rings of anthraquinones. Another interaction is present between the MOF and the hydroxyl groups of anthraquinone through hydrogen bonding. All samples were effectively separated. However, the rate of adsorption was hindered beyond pH 9.5. The pH value plays a pivotal role in the separation process.

### 5.5. The Preparation of Thin Films

Different MIL-100s (Al, Cr, Fe) were synthesised by MW-assisted hydrothermal reactions at temperatures ranging from 130 to 210 °C making it a green synthetic procedure [79,133]. The MIL-100 (Al) was prepared in the presence of acetic acid to act as the blocking agent for the growth of the crystal [134]. The formation of submicrometric crystals is pH dependent: pH 1–3 produced MIL-96 (Al) consisting of Al-chain, pH 0.5–0.7 produced Al-trimer MIL-100 (Al); and pH 2–4 formed inorganic chains and clusters. The porosity of the crystal was low because of crystal impurities. MIL-100 (Cr) was prepared devoid of the formation of MIL-96 (Cr) as an impurity with low crystallinity when prepared in ethanol. However, the particle sizes were larger in the aqueous phase. MIL-100 (Fe) was prepared with better crystallinity in both water and ethanol, forming carboxyl groups at the surface of the crystals. The size of the crystals was reduced when synthesised in pure water or ethanol instead of the water–ethanol mixture because of the presence of impurities, amorphous solids, or the blockage of pores owing to the coordination of ethanol and metal sites. MIL-100 (Fe) and MIL-100 (Cr) nanoparticles were used for the synthesis of transparent thin films through deposition by the dip-coating technique. High-quality ellipsometry films with optical properties with thicknesses of 40 nm and 60 nm were fabricated using both MOFs. The thickness of the thin films was manipulated by depositing several layers. The refractive index of the fabricated film was enhanced thanks to the condensation of water molecules within the porous network. The thickness of the film was decreased owing to stress created by the condensation of water capillaries within mesopores. The mesopores of MIL-100 (Fe, Cr) are weakly hydrophilic with the distribution of large pores. Nevertheless, MIL-100 (Al) formed a nonhomogeneous covering of wafers on silicon and was not suitable for the fabrication of optical quality films.

### 5.6. Supercapacitor Materials

**Figure 7:**
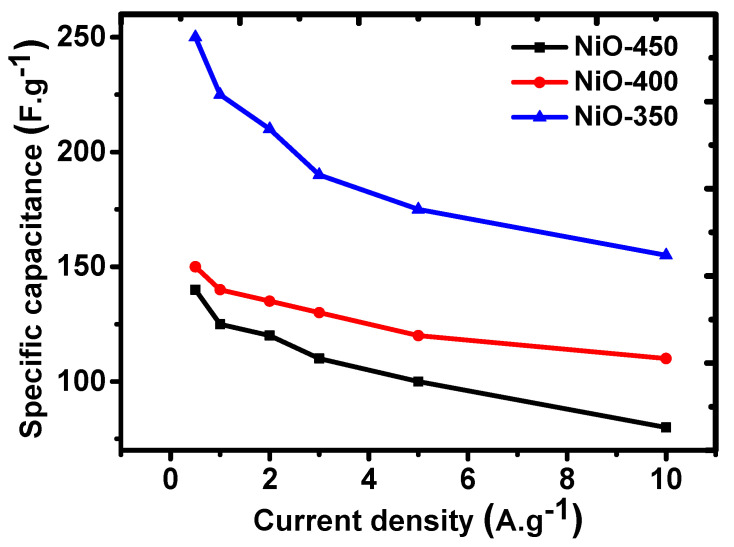
Specific capacitance using the MW-assisted synthesis of NiO nanoparticles.

A MOF with pillared layers of Ni(bdc)(ted)0.5 (bdc = 1,4-benzenedicarboxylic acid, ted = triethylenediamine) was designed under MW irradiation, from which NiO nanoparticles were obtained by annealing the MOF at different temperatures [135]. The growth of NiO particles was enhanced by increasing the calcination temperature without affecting their morphologies. The specific surface area of electrodes in supercapacitors was augmented by micropores, resulting in the amplification of the capacitance. Macropores reduced the distance required for the diffusion of ions and also of stored ions, while the diffusion pathway of ions was improved because of the mesopores, which in turn amplified the electrochemical output [136]. Thus, the mesopores of NiO-350 exhibited the highest specific capacitance thanks to the presence of abundant electroactive sites, as shown in Figure 7. The penetration of electrolytes through pores enhanced the rate of ion transport, and the conductivity of the electrode was increased thanks to the nanosize.

### 5.7. Materials for Lithium–Sulphur Batteries (LIBs)

Mn-MOF (Mn-Bpdc) was prepared under MW heating, which was later converted to MnO/C porous nanocomposites and used in the sulphur cathode surface as the shuttle surface layer of a Li/S battery for improving the performance, as shown in Table 2 [137]. The reactivity of biphenyl-4,4′-dicarboxylic acid was enhanced thanks to the interaction of hydroxyl groups with microwaves [79]. Mn-MOF crystallised as a triclinic space group, where DMF molecules and Bpdc ligands asymmetrically linked to the Mn_4_ cluster. The annealing of the MOF at 700 °C formed MnO/C nanocomposites. MnO formed 40 nm nanoclusters on the surface. The substrate could easily access active sites, which was an asset for nanocomposites to act as the heterogeneous catalyst. Further, d–spacing is contributed by the carbon-encapsulating MnO nanoparticles. Amorphous carbon is converted to a disordered graphitic carbon through partial transformation to form the crystal plane of graphitic carbon [138]. Nanocomposites were used for the fabrication of a shuttle-suppressing layer. Polysulfides were bonded to MnO/C nanocomposites to form the shuttle-suppressing layer and demonstrated a higher retention capacity. A higher potential was obtained as compared with electrodes coated by GO or a simple basic electrode. These electrodes exhibited lower hysteresis thanks to their high conductivity and energy efficiency. The onset of the reduction peak was shifted to a higher voltage, while the oxidation peak was shifted to lower voltage in comparison to the other two electrodes. The cyclic stability of the lithium-ion battery was augmented in that MnO acted as the catalyst and increased the redox process by decomposing Li_2_S/Li_2_S_2_ [139,140]. The soluble lithium polysulfides were adsorbed by the nanocomposite layer, thereby preventing them from being dissolved in the electrolyte. The process of charge transfer was augmented by decreasing the resistance to the charge transfer, and the kinetics of the electrochemical reaction was boosted.

## 6. Future Perspectives and Conclusions

The different routes of the development of MOFs through MW-assisted synthesis, kinetics, and the application of the synthesised MOFs have been reviewed. MOFs were synthesised using organic linkers and inorganic metals under MW heating; this process required considerably less time than conventional methods would. This review focused on the advantages of the MW process: short reaction rates, high yields, the selectivity of phases, and the formation of nano-size crystals. MW-synthesised MOFs catalysed the synthesis of cycloaddition products and different oxidation reactions. Cyclic carbonates were synthesised using different MOFs by customising their pore sizes, chemical states, and dimensions. MOFs participate in different reactions where CO_2_ is used. Transition metal centres are appropriate for the synthesis of MOFs thanks to the formation of rigid geometries and the fast growth of crystals under MW. The defects of MOFs can be effectively manipulated during MW-assisted synthesis. These defects can be enhanced as desired for converting the MOF to heterogeneous catalysts with superior catalytic activities. MOFs formed by MW heating exhibit better structural properties than those synthesised by conventional methods. DUT-52 and Ln (MOF) exhibited photoluminescence, which can be further investigated and used as sensors.

Different methods of MW synthesis have been studied. The solvent-free synthesis is a green method, while the dry-gel conversion method has the advantage of solvent reuse. Co-MOF was synthesised using MW under solvent-free conditions. The main criteria for MW irradiation are the presence of heating spots, which are present in solvents. Thus, the essential requirement for this process to be conducted under solvent-free conditions is for the metal to consist of heating spots. Although cobalt has been identified as a metal containing heating spots, other metals should be investigated for the presence of heating spots for conducting solvent-free MW synthesis. The solvent reuse technique in the dry-gel conversion decreases the use of solvent and contributes to environmental safety. Because the use of solvent is mandatory for the synthesis of specific MOFs, this technique is appropriate, which releases minimal solvent to the atmosphere. However, there is a scope for the discovery of other novel techniques for the synthesis of MOFs with minimal solvent loss. The MW-assisted ball-milling process is an effective method as the speed of the reaction can be enhanced and the reaction pathway can be altered. Thus, it can provide a new synthetic route for the preparation of MOFs.

A few MOFs synthesised under MW exhibited effective adsorption properties for gases, such as CO_2_ and CH_4_, which have the potential to be used for environmental cleaning. The adsorption of drugs is a promising property of MOFs in the biomedical field. The adsorption property of MW-synthesised MOFs can be elaborately studied in the future by manipulating and tuning their pore sizes. Metal-based nanocomposites obtained by pyrolysis from MW-synthesised MOFs also exhibited good adsorption properties, depending on their thickness. Thus, there remains ample scope for the preparation of various nanocomposites from these MOFs. The adsorption of gases depends on the geometry of the MOF. The synthesis parameters using MW, such as temperature, modulators, and solvents, dictate the adsorption capability of MOFs. Future work can improve the adsorption capability of MOFs obtained by MW synthesis under controlled conditions. The process can be scaled up and used for industrial purposes.

The activation process of MOFs using MW has also been elaborated. This is a cheap and lucid method for removing the trapped and coordinated solvent molecules within the MOF structure. The analysis of the dielectric loss and dissipation factor of a specific MOF would reflect the effect of MW activation on similar MOFs.

MnO/C nanocomposites derived from Mn-MOF exhibited excellent results from their being used as a coating on electrodes in Li–S cells. The prospect lies in the examination of effective nanocomposites from MW-synthesised MOFs. The synthesis of uniform nanostructures under MW heating can be used for the synthesis of various porous substances to be used for electronic appliances. MW-synthesised MOF-derived metal composites were used for the photocatalytic decomposition of compounds, such as methylene blue, which demonstrated excellent catalytic activity thanks to increased light absorption and a reduction in the recombination of charge carriers.

The controlled synthesis of γ-CD MOF was carried out within a short reaction time, which showed effective adsorption properties for fenbufen, a drug candidate. Hence, MOFs are potential candidates for applications in drug delivery. MOFs have been successfully used as a pseudostationary phase for the removal of anthraquinones by the capillary electrophoresis process, which can be further studied.

The kinetics of MW absorption provides an insight into the basic understanding of MW absorption, which opens up a field in material science for searching for new materials capable of MW absorption. The most significant challenge of using MW synthesis is the production of large crystals. The crystal size is always smaller than that produced by conventional methods. However, other aspects, such as a short reaction time and a faster rate of nucleation, make the MW process attractive. Hence, the synthesis of larger crystals through crystal engineering by controlling the desired parameters using MW is demanding in the field of material science. MW-based synthetic reactions should be scaled up in the future for large-scale applications.

## 7. Conclusions

The MW-assisted synthesis method of MOFs has a higher yield, good reproducibility, and faster heating and kinetics than the conventional synthesis methods, such as solvothermal synthesis and high-pressure hydrothermal synthesis. Moreover, the morphology and size of the synthesised MOFs can be controlled under MW. MOFs can be activated under MW irradiation for better performance. Solvent molecules present with MOFs can be desolvated within a short time span. Solvent-assisted MW activation is another feasible technique that can have potential applications in the future for the synthesis of thermally unstable MOFs. The size of the synthesised crystals can be manipulated by optimising the MW conditions. Thus, MOF crystals of desired sizes can be obtained as required for multiple applications. MOFs obtained through MW-assisted synthesis have the capability to effectively resist water and effectively capture carbon dioxide, even under humid conditions. Because MOFs prepared through MW possess multiple improved properties, they will be promising candidates for different industrial applications in the future. Additionally, the MOF synthesis time is highly reduced, which has a positive impact on industrial and large-scale applications. MW-assisted synthesis requires minimal solvent and less heating time, thus reducing environmental concerns, and can be a promising alternative in the future.

## Figures and Tables

**Table 1:** The elative crystallinity of MIL-53(Al).

MIL-53(Al)	**Absorbed Power (W)**	**Relative Crystallinity (1/FWHM)**
	269	5.20
	682	4.31
	945	4.76
	1739	5.17
	3819	4.46

**Table 2:** The summary of the electrochemical performance of the MOF-derived MnO/C hybrid structure.

**MnO/C Nanocomposite**	**Initial Discharge Capacity** **(mAh g^−1^)**	**Capacity Retention** **(%)**	**Capacity Fading** **(% per Cycle)**
	851.6	81	0.189

## Data Availability

Not applicable.
